# Supplementary material for: The functional role of visual information and fixation stillness in the quiet eye
Source: PLoS One. 2023 Nov 6;18(11):e0293955. doi: 10.1371/journal.pone.0293955 (PMC10627465; doi:10.1371/journal.pone.0293955)
Supplement: Attachment — Submitted filename: Responses to comments.docx [file pone.0293955.s002.docx]

Additional Editor Comments:

One of the reviewers (Reviewer #2) remains very concerned that there are fundamental issues related to data collection or analyses in the experiment, as evidenced by the large number of trials with no quiet eye periods. Can the authors reconcile their results with results of studies cited by the reviewer in which the quiet eye occurred with far more regularity? For example, is there something fundamentally different with the eye tracker, the methodology/threshold, the task, or the participant population that could account for these differences? Is it possible to vary the threshold or the method of detection of the quiet eye to see whether that makes a difference?

In addition to the critical issue raised by reviewer #2, please provide a reference or references around the "75ms change in quiet eye duration" used in the study.

Reviewer #1: Thank you to the authors for addressing my comments. My last comment is regarding the selection of the 75ms change in quiet eye duration. Can the authors add some references for the reader to identify the previous literature mentioned in their edits?
Thank you for the opportunity to review this manuscript.

Response: Added, thanks (line 160).

Reviewer #2: Review for PONE-D-23-08839R1

I appreciate your response to my earlier comments. However, your response regarding the lack of fixations in about half of the trials was insufficient. Henceforth, my focus will be solely directed towards this specific matter, with the intention of explaining my substantial concerns regarding the methodology adopted in the measurement of Quiet Eye.

Other studies have utilised more stringent spatial dispersion measures than those outlined in the present manuscript. The Tobii 2 glasses that were used in Vincze et al. (2022), for example, use an automated detection method (so fixations were not “manually coded”, as you suggest) and have a precision of 0.05 ° at 1.5 m (reaching 1.1 degree only in extremely poor lighting conditions). Despite these strict requirements for fixations, the measurement of QE was accomplished at a lower data loss rate in the literature (e.g., 1% in Vincze et al., 2022; 6,1 % Klosterman et al., 2018).
Moreover, in some of the aforementioned studies, the task's level of dynamism and speed were significantly higher (e.g., table tennis in Vincze et al.,) I mention all of this because we would expect higher data loss rates in a scenario with stricter precision and more/faster movement than those described in the present study.
Therefore, I do not understand how the high rate of data loss could be adequately explained by a strict definition of fixation alone. Consequently, I have concerns regarding any other factors that may have impacted the measurement process. As a result, I cannot recommend this work for publication in the present form as it does not sufficiently clarify this issue.

Klostermann, A., Kredel, R., & Hossner, E. J. (2018). Quiet eye and motor performance: The longer the better?. Journal of Sport and Exercise Psychology, 40(2), 82-91. doi: 10.1123/jsep.2017-0277
Tobii. (2022). Tobii Pro Glasses 2 Data Quality Test Report. Accuracy, precision, and data loss under controlled environment.
Vincze, A., Jurchis, R., & Iliescu, D. (2022). Quiet eye facilitates processing complex information in elite table tennis players. Visual Cognition, 30(7), 506-516.

Response: Thanks, yes we appreciate that there are a lot of zero values and understand the reviewer digging deeper into this issue. We are confident that the zero values are a legitimate aspect of the data and not some kind of error. To expand on our previous response to this issue:

1. We used a novice population who have typically short quiet eye durations. Novice participants can be quite bad at controlling their attention and may often have no quiet eye at all. The comparison to the Vincze et al. paper with elite sports people who have much better attentional control is therefore somewhat problematic. Indeed, the most consistent finding in QE-related research is this between group difference in QE duration between experts and novices (See Lebeau et al., 2016 meta analysis). As the reason for this difference is related to better attentional control, it is not unexpected that QE detection would be lower in a non-experienced sample population who do not know where to best focus attention (either in terms of duration or location).
2. You mention the spatial dispersion values in other studies, but it is not simply the spatial dispersion criteria that is the issue. It is the fact that because we are using VR we are able to precisely locate both the location of the ball in 3D space and the intersection point of the gaze vector in 3D space, so in addition to requiring the gaze to be stable within a certain spatial dispersion, this must also occur with the gaze staying on the target location (the ball) as well. Our point about the manual coding is that there is a lot more ‘flex’ in that method which allows the fixation to continue if gaze moves slightly, but still remains (functionally) near the ball.

Whereas, with our automated method the determination of this position is more stringent and the quiet eye period would end when gaze and object do not overlap. The automated method in VR deals with issues around the vestibulo-ocular reflex that head mounted eye trackers cannot deal with without some additional head tracking mechanism (i.e., eyes might be stable in relation to the head but point of gaze might be moving, which would be a continuation of a fixation for head mounted eye trackers, but not in VR). The combination of the strict fixation detection algorithm, with the strict matching of the gaze vector to the ball position is the reason why our method resulted in more zero durations than most. We do acknowledge in the paper that our values are probably not comparable to other studies for this reason.

3. A key point that we would like to reiterate as researchers who have adopted both methods, is that zero quiet eye durations are not an error or an indication that something has gone wrong. As such the term ‘data loss’ is problematic. We contend that it is not that we were unable to detect gaze behaviour that was happening, but that this behaviour was not present *to the degree of precision that we have set*.

Additionally, it is incorrect to say: ‘regarding the lack of fixations in about half of the trials’, because, what we show is that there just isn’t a stable fixation *in the right place at the right time* to register as our definition of a quiet eye. A zero quiet eye is a totally legitimate behaviour and may mean the participant has focused near the ball, but not exactly on it, or they looked at it but their eye was not stable. Or that they barely looked at it. All of these things result in a zero quiet eye being recorded but are not ‘data loss’. Also, this outcome occurred on 35% of trials, which means that total novices do not execute a quiet eye on 1 in 3 trials, which seems entirely reasonable if the quiet eye is a measure of expertise and optimal attentional control.

4. While we appreciate the comments and pointers to the Vincze et al. paper, this is not the most appropriate comparator for a couple of reasons. Firstly, it is not a self-paced task, so the quiet eye is a tracking gaze of a moving target. This is in itself a problem when it comes to defining QE across tasks (and something our team has also wrestled with in the past when computing QE variables for tracking tasks). Secondly, in the Vincze et al. paper it is not stated that the fixation was actually matched to the ball’s location (From the Methods section of the paper – “QE was operationalized as the final fixation before the athlete initiates a critical movement. The fixation has to last at least 100 ms and not deviate more than 3°”). Any steady gaze (of >80ms) to any location would have been calculated as a QE, which is a much less stringent than our definition which reflects the fixation to a specific location in time *and* space. It is almost inevitable that there would have been some kind of fixation before movement initiation in the Vincze study, so there would be very few zero values (despite this being a more dynamic task). However, from the description provided in the Methods of the paper, this fixation could have been on the coach’s face, body, arm, the ball, the table, or somewhere in the space between. If instead the authors had matched the gaze vector to the actual location of the ball in 3D space (very difficult with head mounted eye trackers but easy in a VR headset) this would have resulted in a much stricter criterion for the quiet eye and likely many more zero values.
5. The quiet eye calculation method that we used is indeed much more similar to the Klosterman paper, but this comparison is still somewhat problematic. Firstly, Klosterman et al experimentally cued the quiet eye durations of the participants using auditory tones and varying stimulus onset delays. So, they were controlling the durations, which is not comparable to what we did. Secondly, they trained the participants to do this, so again they were not really novices in the throwing task. In addition, throwing a ball is a common skill, whereas golf is much more specialised and unnatural and we specifically recruited novices. Thirdly, again this is not a comparable task because the target appears at a variety of locations, so you can’t really throw a projectile at it without looking at it. By contrast, during putting you know the location of the target, so a quiet eye becomes an attentional “choice” and might not be activated (or even understood as a solution to performance feedback). Indeed, as we showed in this very study, you can putt effectively without having to look at the ball at all (occlusion conditions). So, it is entirely possible that participants would not have had a quiet eye sometimes (especially novices).

Given these points, we hope the reviewer will consider that these two studies are really not comparable to what we have done, and that the differences we have pointed out are where the much higher percentage of zero values have come from. Most quiet eye studies present averaged quiet eye values and have not provided the raw data in open repositories, so it is hard to know how often novice performers have zero quiet eye values in these studies. To reiterate, zero values are not ‘data loss’, they are just when a participant did not execute a stable fixation on the ball at the right time, something that would be a common occurrence in our novice population.
