# Supplementary material for: The functional role of visual information and fixation stillness in the quiet eye
Source: PLoS One. 2023 Nov 6;18(11):e0293955. doi: 10.1371/journal.pone.0293955 (PMC10627465; doi:10.1371/journal.pone.0293955)
Supplement: Attachment — Submitted filename: Rebuttal.docx [file pone.0293955.s003.docx]

Additional Editor Comments:

Thank you for the extensive response to the reviewer's comments. Can you please indicate what has been changed/added in the manuscript to address the reviewer concerns (perhaps by highlighting these changes or by using a different font). Much of this discussion can perhaps be incorporated in a separate section in the paper perhaps entitled "methodological considerations with the quiet eye" or something like that. The journal readership will benefit from this discussion.

Response: Thanks. As you saw from the previous set of responses we think the reviewers comments were based on a limited understanding of this literature in this area, so did not warrant any substantive changes to the manuscript. We tried to justify this in our detailed responses in which we showed that the reviewer’s comparisons to other studies were not warranted.

In response to this additional request, we have added a paragraph to the discussion to outline some of these methodological issues and how our paper compares to previous work in this area (lines 658-667).
